# Unexpected appetitive events promote positive affective state in juvenile European sea bass

**DOI:** 10.1038/s41598-023-49236-5

**Published:** 2023-12-12

**Authors:** M. V. Alvarado, A. Felip, F. Espigares, R. F. Oliveira

**Affiliations:** 1 Integrative Behavioural Biology Group, Instituto Gulbenkian de Ciência, 2780-156 Oeiras, Portugal; 2 Fish Reproductive Physiology Group, Institute of Aquaculture Torre de la Sal, IATS-CSIC, Ribera de Cabanes, 12595 Cabanes, Castellón Spain; 3 ISPA-Instituto Universitário, 1149-041 Lisbon, Portugal; 4 Champalimaud Neuroscience Programme, Champalimaud Centre for the Unknown, 1400-038 Lisbon, Portugal

**Keywords:** Behavioural ecology, Animal behaviour

## Abstract

Some animal species exhibit considerable physiological and behavioural alterations in response to captivity. It has been hypothesized, but rarely tested, that such changes reflect a negative affective state that is associated to this specific context. In the last years, judgement bias measures have emerged as reliable indicators of animal affective state, under the assumption that individuals in a negative affective state are more likely to evaluate ambiguous stimuli as negative and display therefore pessimistic behaviours. Here, we have developed a judgement bias task for juvenile European sea bass (*Dicentrarchus labrax*) aiming to measure optimism/pessimism in this marine species, which have previously been reported to show important dysregulations in captive settings. Our results show that juvenile sea bass exhibit a considerable bias towards pessimistic behaviours in laboratory settings. Furthermore, juveniles that received an unexpected positive event during the judgement bias test displayed more optimistic responses toward ambiguous stimuli as compared to control fish, indicating a positive change in their affective state induced by the appetitive experience. These results reveal a direct interaction of the internal affective state with decision-making processing under ambiguity in juvenile European sea bass, highlighting therefore the potential of judgement bias tests as a tool for the advancement and improvement of our understanding of welfare in finfish aquaculture.

## Introduction

The study of emotions or affective states in non-human animals has been causing controversy since Darwin’s 1872 monograph “The Expression of Emotions in Man and Animals”, in which he considered that behavioral “emotional” expression is recognizable not only in mammalian species such as chimpanzees, dogs and cats, but also even in insects^1^. A frequent point of confusion in arguments about affective states in animals is the anthropocentric conceptualization of emotion, which is based on the existence of feelings (the human subjective perception of emotions). This conventional view implies that emotions cannot be studied in any organism other than *Homo Sapiens*^2^, since the existence of feelings, and consequently of emotions, could only be assessed by verbal report. On the contrary, a recent approach aiming to study of affective states in animals has conceptualized emotions as internal states that are characterized by the expression of externally observable behaviours and accompanying physiological responses^3^. This framework does not depend on vocal reports or anthropocentric homologies since it is based on the study of general features of emotions (aka *emotion primitives*) that apply across phylogeny (e.g. persistence, scalability, valence, generalization)^3–5^. This approach opens the way for analysing behaviours that provide evidence for such *emotion primitives* across phylogeny and, consequently, for the establishment of behavioral assays for the study of affective states in non-human animals.

A consistent finding from human psychology is that the valence of an individual’s affective state (which has been considered an evolutionary building block of emotions or an *emotion primitive* as discussed above) might influence cognitive processes such as judgement^6^. For instance, individuals experiencing both depression and anxiety often exhibit a tendency to make negative judgements about ambiguous situations, reflecting an enhanced expectation that negative events will happen to the individual^7,8^. This association between affective state and decision-making has attracted attention from many researchers in the last years and experimental paradigms to assess judgement bias have been developed for a broad range of species e.g.^9–12^, revealing that these behavioral measures indeed provide reliable indicators of the valence of emotional states in animals^13–15^. Judgement bias in non-human organisms has been conceptualized as a decision-making process in which some individuals interpret ambiguous stimuli as positive (optimism) and others as negative (pessimism). Most judgement bias studies have been focused on the modulation of decision-making under ambiguity by situational or contextual factors (e.g. environmental enrichment) that are expected to affect the affective state of the animals e.g.^16–18^. Judgement bias has therefore been traditionally considered as a transient condition (i.e. a behavioral state). However, a recent study in zebrafish has shown that judgement bias also has a relatively stable individual component over time and thus can be also considered as a personality trait, with different individuals expressing different judgement bias phenotypes (i.e. optimists *vs.* pessimists)^19^. Furthermore, evidence for a trait-like influence on judgement bias responses has been also reported in mammals^20,21^ and birds^22^.

The fact that judgement bias tests have emerged as one of the most valid tools in measuring affective states in animals is of great significance for the assessment and monitoring of animal welfare in farm and laboratory settings. Some animals that are brought into these settings undergo profound physiological and behavioral changes in response to captivity^23–25^. In fact, many features of the captive environment, which include forced proximity to humans, exposure to artificial lighting, and lack of environmental enrichment, have been shown to induce an overactivation of the hypothalamic–pituitary–adrenal stress axis^26–28^. It can be therefore hypothesized that such environmental stress may lead captive animals to display a negative judgement bias, as has been observed in rats exposed to chronic restraint^20^, which would reflect a negative affective state. In this study, we have developed a judgement bias task specifically adapted for juvenile European sea bass (*Dicentrarchus labrax*) aiming to measure optimism/pessimism in this marine species. European sea bass exhibit high responsiveness in captive environments, showing important dysregulations associated to human proximity, routine husbandry practices and handling^29–31^. This makes European sea bass an interesting, although challenging, model to study decision-making under ambiguity in a captivity context. We first characterized judgement bias in juvenile sea bass by measuring individual responses to ambiguity in a homogeneous sea bass population as well as its correlation with other behaviours, as measured by open field with shelter and novel object tests. Then, and considering the recently reported effect of unexpected positive experiences on the affective state in non-human animals^32,33^, we analysed the effect of unexpected appetitive events on decision-making under ambiguity, which are expected to induce positive changes in the affective state of the juveniles.

## Results

### Juvenile sea bass display pessimistic-like behaviours in laboratory conditions

**Figure 1:**
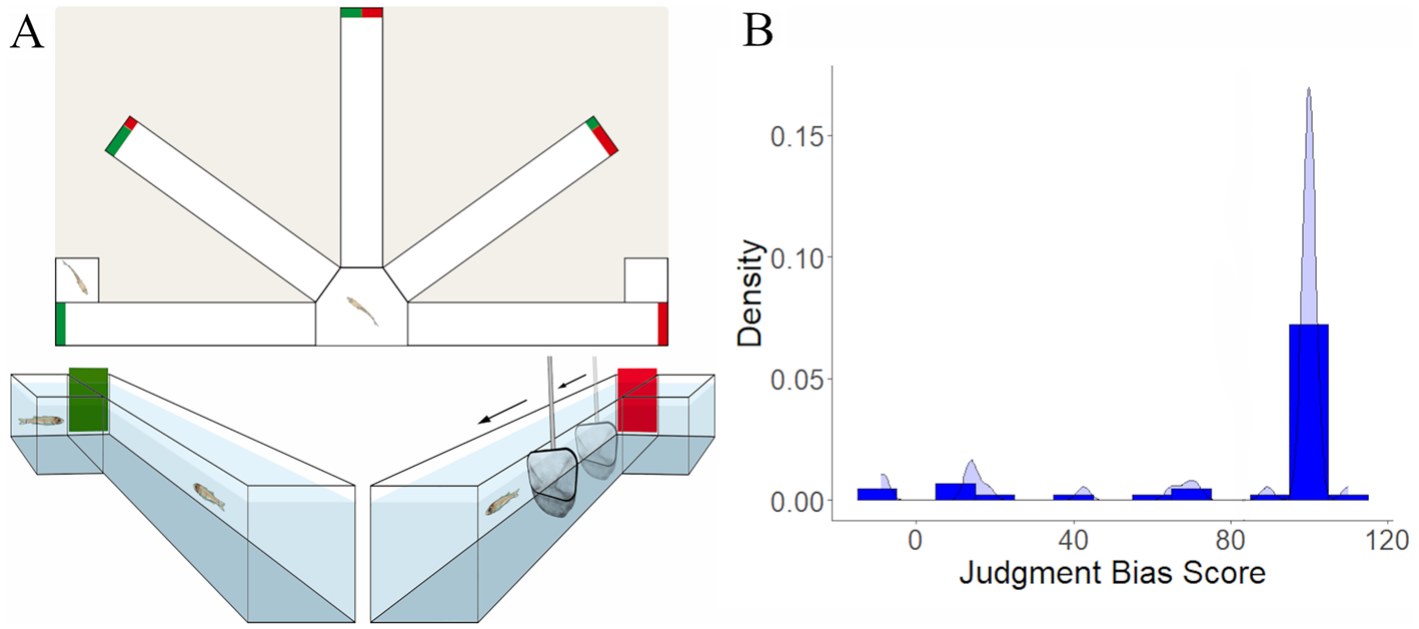
Behavioral characterization of judgement bias in juvenile European sea bass. (**A**) Diagram of the half radial maze highlighting the two reference arms (positive/rewarded (P; social reward/ exposure to a conspecific) and negative/aversive (N; punishment/ chasing with a net)) that were used in the judgement bias test (JBT) for juvenile sea bass. Once juveniles are able to discriminate between P and N arms, only their responses to an ambiguous arm—which is located midway between the two reference arms (90°) and contains a mixed coloured card (1:1)—were tested. (**B**) Illustration of the combination of the frequency distribution of the judgement bias score (JBS) among juveniles that completed the judgement bias task (n = 43) with the Kernel density estimation, which calculates the probability density of JBS. Drawing of diagram in (**A**) by Iara Chapuis.

Our results show that juvenile sea bass fell into a skewed normal JBS-frequency distribution characterised by a very long left tail with optimistic-biased fish and a right modal extreme with pessimistic-biased fish (Fig. 1B). Specifically, 32 juveniles (74.4% of the experimental population) did not enter the ambiguous arm and exhibited a JBS ≥ 100 (JBS = 100.31 ± 0.31). A JBS ≥ 100 indicates that individuals are treating the ambiguous cue like the negative one, thus suggesting a robust pessimistic judgement bias. On the contrary, only 11 juveniles (25.6% of the experimental population) enter the ambiguous arm (JBS = 34.53 ± 10.35), showing more optimistic responses as compared to juveniles that did not enter the arm. Considering these results, optimistic and pessimistic thresholds in our experiment were determined based on the number of juveniles that entered or not the ambiguous arm.

Our results indicate that the experimental population showed only a modal extreme with pessimistic-biased fish, with three quarters of the scored individuals exhibiting a JBS ≥ 100.

### Judgement bias is paralleled by differences in exploratory and boldness behaviours in juvenile sea bass

**Figure 2:**
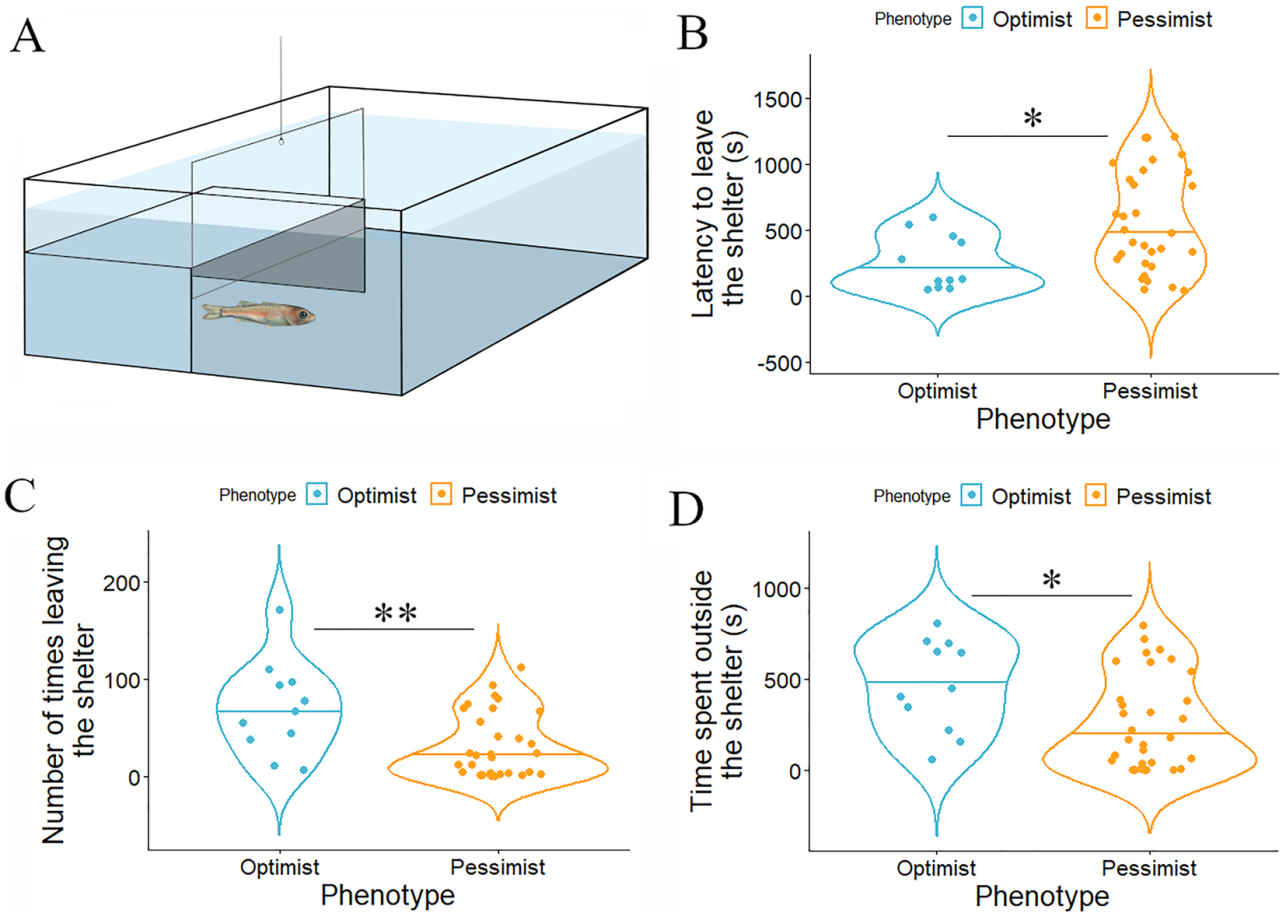
Behavioral differences between optimistic and pessimistic juveniles (n = 11 and n = 32, respectively) in the open field test with shelter (OFT): (**A**) Diagram of the OFT; (**B**) latency to leave the shelter for the first time (s); (**C**) number of times leaving the shelter; and (**D**) time spent outside the shelter (s). Asterisks indicate significant differences between experimental groups. Drawing of diagram in (**A**) by Iara Chapuis.

The presence of a small percentage of fish showing more optimist-like behaviours presented an opportunity to compare specific behaviours between juveniles that presented different responses to ambiguous stimuli. Scored individuals were then tested in two behavioural paradigms that aim to obtain measures of exploratory and boldness behaviours [open field test with shelter (OFT) and novel object test (NOT)]. In our study, we examined several measures of activity and location of optimistic and pessimistic juveniles during the whole OFT (Fig. 2A) trial (20 min). Our results show that, upon opening the door of the shelter, optimistic juveniles rapidly entered the open field arena as compared to pessimists (Fig. 2B; two-sided unpaired t-test, t_41_ =  − 2.416, *p = 0.020; Cohen’s d =  − 0.84, 95% CI = [− 1.54, − 0.13]). Furthermore, optimistic juveniles left the protection of the shelter a higher number of times (Fig. 2C; two-sided unpaired t-test, t_40_ = 2.841, **p = 0.007; Cohen’s d = 1.02, 95% CI = [0.29, 1.74]) and, consequently, spent more time in the open field arena (Fig. 2D; two-sided Mann–Whitney rank-sum test, T = 328.0, *p = 0,017; Cohen’s d = 0.81, 95% CI = [0.10, 1.51]). These results indicate that optimistic juveniles exhibit a high propensity to explore novel environments than pessimistic juveniles.

**Figure 3:**
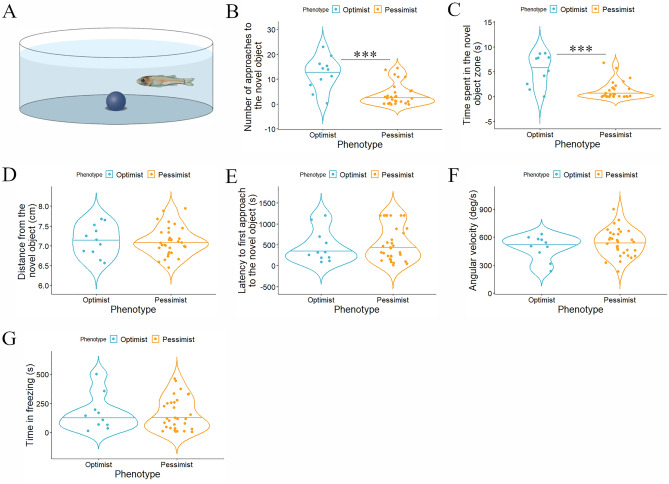
Behavioral differences between optimistic and pessimistic juveniles (n = 11 and n = 32, respectively) in the novel object test (NOT): (**A**) Diagram of the NOT; (**B**) number of approaches to the novel object; (**C**) time spent in the novel object zone (s); (**D**) distance from the novel object (cm); (**E**) latency to first approach to the novel object (s); (**F**) angular velocity (deg/s); and (**G**) time in freezing (s). Asterisks indicate significant differences between experimental groups. Drawing of diagram in (**A**) by Iara Chapuis.

In our study, we examined several measures of novel object examination and mobility of optimistic and pessimistic juveniles during the whole NOT (Fig. 3A) trial (20 min). Our results show that optimistic fish approached a higher number of times the novel object (Fig. 3B; two-sided unpaired t-test, t_37_ = 3.803, ***p < 0.001; Cohen’s d = 1.62, 95% CI = [0.82, 2.40]), also spending a higher portion of their time next to it (Fig. 3C; two-sided unpaired t-test, t_36_ = 4.329, ***p < 0.001; Cohen’s d = 1.86, 95% CI = [1.01, 2.69]). However, no statistically significant differences were found between both experimental groups in the distance from the novel object (Fig. 3D; two-sided unpaired t-test, t_41_ = 0.203, *p* = 0.840; Cohen’s d = 0.07, 95% CI = [− 0.61, 0.76]), latency to first approach to the novel object (Fig. 3E; two-sided Mann–Whitney rank-sum test, T = 226.5, *p* = 0.675; Cohen’s d =  − 0.18, 95% CI = [− 0.87, 0.50]), angular velocity (Fig. 3F; two-sided unpaired t-test, t_40_ =  − 0.965, *p* = 0.839; Cohen’s d =  − 0.36, 95% CI = [− 1.07, 0.36]) and time in freezing (Fig. 3G; two-sided unpaired t-test, t_39_ = 0.204, *p* = 0.340; Cohen’s d = 0.04, 95% CI = [− 0.67, 0.75]). Some of these variables suggest that optimistic fish have a higher willingness to engage in risky behaviours in order to investigate novel objects as compared to their pessimistic counterparts.

In summary, our results show that judgement bias correlates with other behaviours, with pessimistic juveniles exhibiting a lower propensity to explore novel environments and a lower willingness to approach novel objects as compared to optimistic fish.

### Judgement bias is not part of a behavioural syndrome in juvenile sea bass

To assess the phenotypic architecture of decision-making in juvenile sea bass we performed a principal component analysis (PCA) based on the correlation matrix between the behavioural measures extracted from the 3 abovementioned assays (JBT, OFT and NOT). PCA on the ten behavioural measures extracted from the videos (see Table 1) identified three principal components (PC; eigenvalue > 1). PC1 (eigenvalue = 3.65, variance explained = 36.5%) showed a strong loading of spatial examination, object examination and willingness to take risk measured both in OFT and NOT, suggesting the occurrence of a behavioural module related to exploration and boldness that only is expressed when individuals are exposed to novel stimuli. PC2 (eigenvalue = 2.00, variance explained = 20.0%) showed a strong loading of mobility measured in the NOT, which may correspond to an anxiety behavioural module. PC3 (eigenvalue = 1.43, variance explained = 14.3%) showed a robust loading of object-orienting measured in NOT, suggesting the occurrence of an activity module (the physical activity of the fish and its propensity to stay either closer or farther from the novel object are reflected in this module). All principal components with eigenvalues ≥ 1 explained a cumulative variance of 70.8%.

**Table 1:** Loadings extracted by principal component analysis from the correlation matrix of behaviours across tests for European sea bass.

Test	Behavioural parameter	Biological meaning of the metric	Principal component loadings ^a^		
			PC1 Boldness/exploration	PC2 Anxiety	PC3 Activity
JBT	JBS	Decision-making under ambiguity	0.294	0.492	− 0.156
OFT	Latency to leave the shelter	Willingness to take risks	**0.687**	0.511	0.101
	Number of times leaving the shelter	Spatial examination	− **0.846**	− 0.344	− 0.141
	Time spent outside the shelter	Spatial examination	− **0.672**	− 0.422	− 0.378
NOT	Distance from novel object	Object-orienting	− 0.078	0.019	− **0.859**
	Latency to first approach to the novel object	Willingness to take risks	0.604	− 0.282	− 0.397
	Time spent in the novel object zone	Object examination	− **0.806**	0.167	0.224
	Number of approaches to the novel object	Object examination	− **0.793**	0.305	0.332
	Angular velocity	Mobility	0.372	− **0.723**	0.314
	Time in freezing	Mobility	0.368	− **0.687**	0.277
		Eigenvalue ^b^	3.653	2.000	1.432
		% Variance explained	36.538	20.002	14.322

Bold type indicates the strongest contributors (coefficient > 0.64) to each principal component (PC).

^a^Correlation between principal components and variable values.

^b^Variance of transformed data used for each component.

These results indicate the JBS does not cluster with any behavioural metric derived from OFT and NOT, and consequently judgement bias cannot be considered as part of a broad behavioural syndrome in juvenile sea bass. Furthermore, a boldness–exploration behavioral syndrome has been found in this fish species.

### Unexpected appetitive events induce positive judgement bias changes in juvenile sea bass

**Figure 4:**
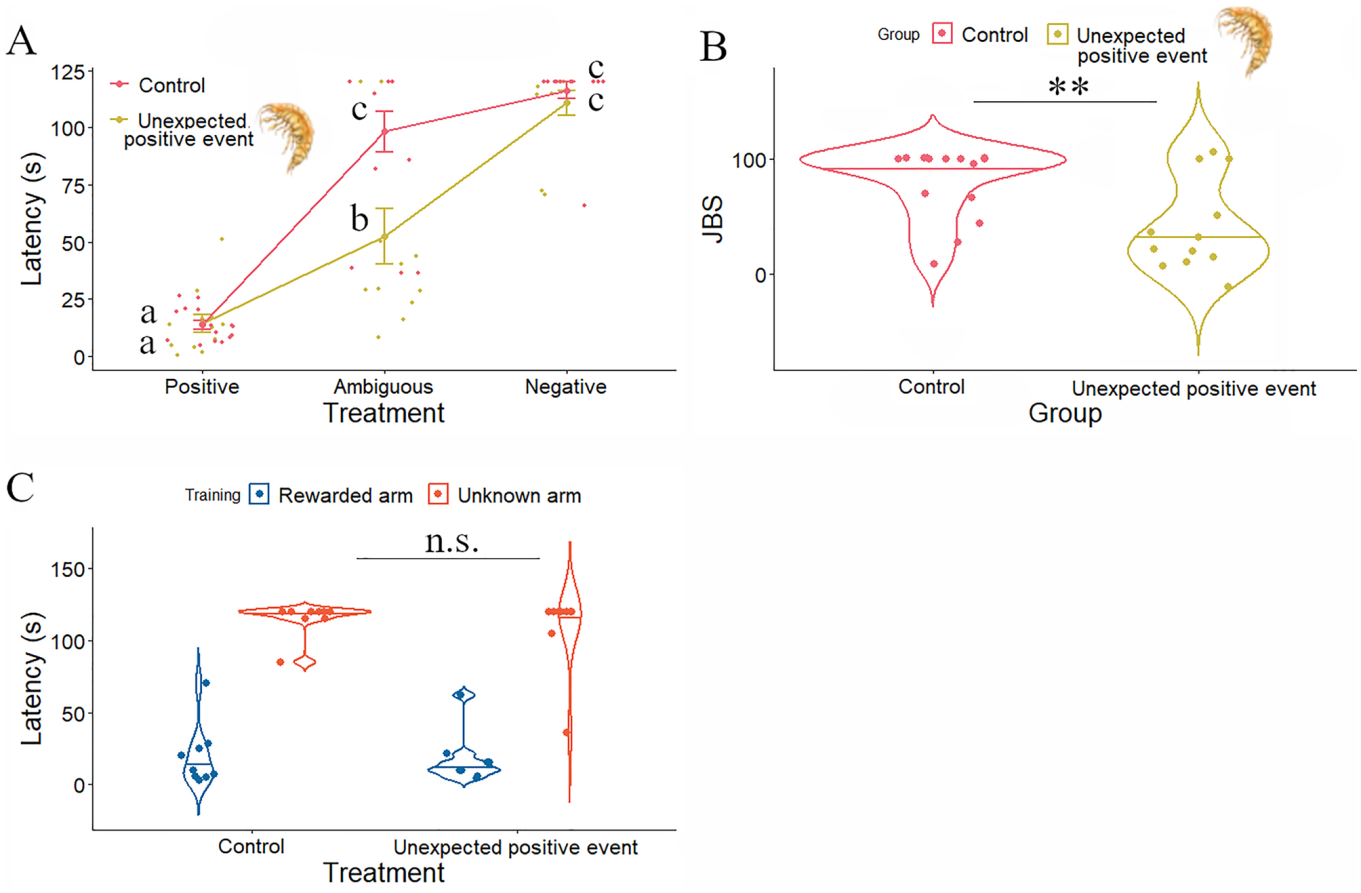
Effects of unexpected appetitive experiences on decision-making under ambiguity. (**A**) Performance of juvenile sea bass receiving or not pretest positive experience (specimen of *Gammarus*) in the judgement bias paradigm (n = 12–15 per experimental group: control and unexpected positive event). Different letters indicate significant differences between the experimental groups for each stimulus (P, N, A) following planned comparisons tests. Data are expressed as mean ± s.e.m.; (**B**) JBS of juvenile sea bass receiving or not pretest positive experience (n = 12–15 per experimental group: no pretest positive event, and pretest positive event). Asterisks indicate significant differences between experimental groups; (**C**) Response of juvenile sea bass receiving or not pretest experience (specimen of fresh frozen *Gammarus pulex*) to a novel stimulus (n = 9 per experimental group: control and unexpected positive event). Drawing of *Gammarus* in (**A**) and (**B**) by Iara Chapuis.

Our results show that juveniles that received no pretest positive experience (i.e. control group) exhibited, as previously described in Experiment 1, a robust bias towards pessimistic-like behaviours (Figs. 4A, B). In contrast, juveniles that received food (*Gammarus*) before making a decision took less time to enter the ambiguous arm as compared to their control counterparts (Fig. 4A; Table 2; planned comparison with two-sided Tukey's HSD, t_61.5_ = 3.239; ***p = 0.0019; Cohen’s d = 1.21, 95% CI = [0.37, 2.03]). The analysis of the JBS yielded similar results, with juveniles that received pretest positive experience exhibiting more optimistic-like behaviours (Fig. 4B; two-sided unpaired t-test, t_25_ =  − 2.946, **p = 0.007; Cohen’s d = 1.14, 95% CI = [0.31, 1.95]). These results show that juveniles that consumed the pretest positive outcome displayed more optimistic response towards ambiguous stimuli than juveniles that received no pretest positive experience.

**Table 2:** Results of the general linear mixed model to assess the effects of stimulus (Positive (P) versus Negative (N) versus Ambiguous (A)), treatment (Control versus Unexpected positive event), and the double interaction among these variables.

Latency				
	F-value	*p* (> F)	Partial Eta^2^	95% CI
Stimulus	F_2,50_ = 139.5647	***p < 0.001	0.85	[0.78, 1.00]
Treatment	F_1,25_ = 4.7517	*p = 0.038	0.16	[0.00, 1.00]
Stimulus × Treatment	F_2,50_ = 3.4769	*p = 0.038	0.12	[0.00, 1.00]

Partial Eta Squared estimates of effect sizes are given for these factors. Asterisks indicate a significant effect.

It could be argued that juveniles that consumed the pretest positive outcome exhibited an enhanced motivation for exploring novel stimuli as consequence of experiencing a higher expectation of subsequent positive events. However, in our study, juveniles tested with a stimulus that is novel in terms of colour and location (i.e. a stimulus that cannot be associated to the trained stimuli) exhibited no difference in the latency to enter the unknown arm (Fig. 4C; Table 3; planned comparison with two-sided Tukey's HSD, t_31.9_ = 0.319; *p* = 0.7515; Cohen’s d = 0.28, 95% CI = [− 0.65, 1.21] ) between the two groups, indicating that pretest outcome consumption do not evoke a general increase in expectation of positive events.

**Table 3:** Results of the general linear mixed model to assess the effects of stimulus (Training versus Novel), treatment (Control versus Unexpected positive event), and the double interaction among these variables.

Latency				
	F-value	*p* (> F)	Partial Eta^2^	95% CI
Stimulus	F_1,16_ = 99.7175	***p < 0.001	0.86	[0.73, 1.00]
Treatment	F_1,16_ = 0.0268	*p* = 0.872	1.67e-03	[0.00, 1.00]
Stimulus × Treatment	F_1,16_ = 0.0851	*p* = 0.774	5.29e-03	[0.00, 1.00]

Partial Eta Squared estimates of effect sizes are given for these factors. Asterisks indicate a significant effect.

Altogether, these data indicate that unexpected appetitive events induce an increase in expectation of positive outcomes only when juveniles are exposed to ambiguous stimuli.

## Discussion

We have developed a judgement bias test for juvenile sea bass that allowed us to assess affective states in this economically relevant aquaculture species for the first time. As expected from other studies on sea bass that suggest a high stress response to captivity conditions^29–31^ we found a potential bias towards a negative affective state in juvenile sea bass exposed to our JBT. The skewed distribution of the JBS in our experimental population characterized by a higher modal extreme of pessimistic-biased juveniles could indeed reflect the occurrence of a contextual factor that induces a massive shift of the behavioral responses of juvenile sea bass towards pessimism. For instance, zebrafish exposed to similar experimental conditions, and even when experiencing a higher salience of the aversive stimulus (i.e. punishment), have been shown to exhibit a bimodal distribution^19,34^, with a significant proportion of the population displaying an optimistic judgement bias. It is plausible, therefore, that some contextual factors inherent to our experimental setting, including handling, social isolation, and/or overreaction to aversive stimuli, may affect the performance of juvenile sea bass in the JBT. In fact, European sea bass exhibit high stress responsiveness in captive environments with important physiological and behavioral dysregulations associated to human proximity and routine husbandry practices (e.g. 64–83% of inhibition of food intake for several days)^30,31^. Such long-term alterations could reflect a negative affective state induced by this specific context, providing therefore a good candidate for explaining the potential bias towards pessimistic behaviours displayed by juvenile sea bass in laboratory settings. Indeed, judgement bias has consistently been reported to be modulated by the individual’s affective state^e.g.^^18,35,36^.

It should be noted that in this study we have used a social reward and not a food reward, which is the most used reward in judgement bias tasks in animals^37^. In our pilot experiments, in which we used 1-mm piece of bloodworm^12^ as reward, no robust learned response to the stimuli (positive and negative) was displayed by juvenile sea bass. Juveniles exhibited difficulties in consuming food rewards during the first training phases, which prevented an accurate acquisition of the available information related to the task. Improving learning acquision of positive outcomes would likely imply a drastic increase of training sessions, leading to a significant augmentation of the assay’s overall duration. Judgement bias tasks require social isolation during training and cue testing^37^, which might make the procedure especially challenging for European sea bass juveniles due to their gregarious nature^38,39^. Training and testing juvenile sea bass individually, without direct contact to its group, could therefore increase their stress levels and/or decrease their willingness to consume the food reward and/or perform the required responses. Therefore, we used a social stimulus (i.e. exposure to a conspecific), instead of food, as a reward. Indeed, social rewards allowed an accurate discrimination between positive and negative stimuli. These data suggest that social rewards are efficient for juvenile sea bass to be individually trained and tested in a judgement bias test.

The presence of a small percentage of juveniles that displayed optimist behaviours in our experimental population presented an opportunity to correlate judgement bias with other behavioral metrics extracted from tasks that are designed to assess specific behavioral traits. Our results show that variability in judgement bias is paralleled by differences in exploratory and boldness behaviours, with pessimistic juveniles exhibiting a lower propensity to explore novel environments and lower willingness to approach a novel object as compared to optimistic fish. These results are in agreement with those found in other species (e.g. pigs and dairy calves), in which pessimistic judgement biases were positively correlated with stronger fearful responses towards novelty^40,41^. From an ecological perspective, a lower willingness to engage in risky behaviours in order to investigate novelty may negatively impact propensities for behavioral innovation^42^ and physiological stress responses^43^ as well as have implications for competitive ability^44^, aggression^45^ and fitness^46^. Interestingly, despite these associations a PCA used to assess the phenotypic architecture of these set of behaviours revealed that decision-making under ambiguity, which was extracted from the judgement bias test, does not load to any behavioral module and, consequently, this behaviour does not cluster with any behavioural metric derived from novelty-related tests. On the contrary, a recent study in zebrafish reported the occurrence of an underlying behavioral syndrome that links the response to ambiguous stimuli with the response to novel objects^19^. The fact that judgement bias does not contribute to the phenotypic architecture of decision-making in juvenile sea bass might indicate that judgement bias metrics in our experimental conditions are affected by the environmental context (i.e. laboratory settings), which temporary disrupts the normal configuration of such phenotypic architecture. Indeed, stress-related environmental constraints have been showed to disrupt an underlying behavioral syndrome in convict cichlid fish^47^. Therefore, these results support our hypothesis that artificial environments might lead juvenile sea bass to a negative affective state inducing, consequently, a pessimistic judgement bias that disrupts the above-mentioned phenotypic architecture. Notably, our PCA analysis also showed novel evidence for a phenotypic link between boldness and exploration (i.e. a boldness–exploration syndrome) in juvenile European sea bass, which has also been observed in other fish species^48–50^. Selection on boldness behaviour in European sea bass could therefore produce indirect correlational selection on exploratory behaviour or vice versa^51,52^, which could have important implications for ecological dispersal in this marine species^49,53,54^.

Given the overall pessimistic judgement bias exhibited by juvenile sea bass in laboratory settings, we decided to test whether a positive change in their affective state could affect their response to ambiguity. Our results show that juveniles receiving an unexpected positive event indeed responded in a positive manner toward the ambiguous stimulus. Unexpected positive events have been reported to induce an abnormal brain function in ventral striatal and orbitofrontal regions in both depression and schizophrenia patients as compared to healthy participants^55^, establishing a direct link between unexpected positive experience processing and the generation of motivational states. A similar relationship between unexpected positive events and the generation of positive affective states has also been showed in non-human animals^32,33^. Therefore, the optimistic responses displayed by juvenile sea bass that consumed a pretest positive outcome indicate an underlying positive affective state induced by the unexpected experience. The fact that consuming the pretest outcome did not evoke a general increase in expectation of appetitive experiences, indicate that it did not simply cause juveniles to become more exploratory. Altogether, these experiments confirmed a direct interaction of the internal affective state with decision-making under ambiguity in juvenile sea bass. These results support the notion that animals have internal states that fit the criteria for defining an affective state or emotion^3,56^. On the other hand, it is also worth noting that the rewarding outcome (i.e. exposure to a conspecific) in our JBT falls within the 'social' functional domain, while the affect manipulation (i.e. food outcomes) is not directly related to this domain but rather to a 'foraging' functional domain. This provides evidence that positive information acquired from different functional domains might be integrated resulting in a general valenced affective state that influences decision-making across contexts^57^. Being able to manipulate an individual’s affective state (e.g. by inducing positive emotional changes) is of great importance in the design of strategies for promoting positive welfare in captive animals. In fact, and although several environmental enrichment strategies have already been applied for improving welfare in farmed fish^58^, the development of judgement bias tests shows considerable promise as a new tool for monitoring affective states in fishes, helping to improve fish welfare in finfish aquaculture.

In summary, our study highlights the fact that juvenile sea bass exhibit an important bias towards pessimistic behaviours in laboratory settings, which can be mitigated by the occurrence of unexpected appetitive events, opening the way to manage negative affective states of an economically important species in aquaculture settings.

## Methods

### Ethics statement

All procedures were performed in accordance with the relevant guidelines and regulations for animal experimentation, reviewed by the Institute of Applied Psychology (ISPA) Ethics Committee and by the institutional Organ Responsible for Animal Welfare (“Órgão Responsável pelo Bem-Estar dos Animais”, ORBEA) and approved by the competent Portuguese authority (Direcção Geral de Alimentação e Veterinária; permit number 0421/000/000/2019). Animals were handled according to the guidelines for animal experimentation established by Spanish Royal Decree (RD 53/2013) and EU Directive (2010/63/EU). All methods are reported in accordance with ARRIVE guidelines (https://arriveguidelines.org) for the reporting of animal experiments.

### Fish and rearing conditions

Juvenile sea bass 90 to 130 dph (0.64–7.15 g in weight) were used in this study. Fertilized eggs were collected from the Institute of Aquaculture Torre de la Sal (IATS, Castellón, Spain) during the spawning season, and egg incubation and larval rearing were carried out following standard procedures for sea bass aquaculture^59^. Fish were maintained under natural photoperiod and temperature conditions (temperature range from 11.05 to 27.6 °C) at the IATS facilities (40° N and 0° E) and fed commercial pelleted food (BioMar, Skretting) of an appropriate size to satiety. Fish were kept in 500L fiberglass tanks, supplied with a seawater dispenser on the top and 4–5 oxygen dispensers in the bottom to aerate seawater (salinity = 37–38‰), at a stocking density < 2 kg m^−3^.

### Experimental apparatus and behavioural procedures

#### Judgement bias test (JBT)

In this study, an already validated protocol for measuring judgement bias in zebrafish^60^ has been adapted to juvenile European sea bass by adding some modifications. This behavioural assay is designed as a Go/No-go task that is accomplished in a half radial arm maze, in which the five radial arms are interconnected through a hexagonal arena (starting box) of 86.9 cm^2^. Each arm is 24 cm long and 5 cm wide, with 8-cm-high side walls. In brief, juveniles are trained to perform either a Go (positive (P) arm) or a No-go (negative (N) arm) response when a specific cue (location/colour cue) is presented signalling the experience a positive event (i.e. social reward/ exposure to a conspecific; Go response) or a negative event (i.e. punishment/ chasing with a net; No-go response). Once juveniles can distinguish between P (Supplementary Video 1) and N (Supplementary Video 2) arms (as indicated by a difference > 40 s in the latency to enter these arms; Latency N – Latency P > 40 s), their response to an unreinforced ambiguous cue (A; an intermediate location/colour cue between P and N) is tested.

The behavioral maze is made of glass and covered with external white PVC sheets. It is equiped with manually operated guillotine doors linking the starting box with each of the arms (Fig. 1A). Each arm contains removable coloured cards that are located at end of the arm and in front of the guillotine doors. The two training (reference) arms (positive (P) and negative (N)) are positioned at 180° from each other and are equipped with full coloured cards (green or red). The three ambiguous (test) arms (near positive (NP), ambiguous (A), and near negative (NN)) are positioned at equidistant angles, each separated by 45° between the two reference arms. In the present study, NP and NN cue testing is omitted from the behavioral procedure since their outputs are not required for the calculation of the judgement bias score (JBS) and the subsequent categorization of individuals along a judgement bias dimension^12,34^. Importantly, omitting NP and NN cue testing results in a shorter test phase. A shorter test phase, and consequently, fewer training trials in this phase, can help to minimize potential confounding effects of stress that might arise from increasing the number of negative outcomes (i.e. punishments) and/or extending the overall duration of the test phase. The ambiguous arm (A) is located midway between the two reference arms (90°) and contains a mixed coloured card (colour proportion of 1 green: 1 red). Researchers operate the guillotine doors from behind a curtain so that they are not visible to the fish during the procedure. Fish behaviour is visualized and recorded through an overhead HP webcam HD 4310 linked to a monitor.

The task consists of 3 phases, which are performed in 3 consecutive days: habituation (day 1), training (2 sessions, day 1 and 2) and test (day 3). Fish are food deprived between phases to minimize excessive handling associated with tank cleaning and water changes. On the day of the experiment, two hours before starting the habituation phase, juveniles are placed in 6L tanks divided by transparent and perforated partitions so that they are partially isolated (i.e. olfactory and visual cues from conspecifics were present) during the procedure. Juveniles are then individually exposed to the behavioural maze for the first time (habituation phase). Each fish is quickly transferred from its isolation tank to the behavioural maze by gently scooping it with a net and then placing the net in the judgement bias apparatus. Each fish is allowed to explore the whole maze for the initial 10 min, since all the doors are opened during this phase. Once the 10 min have elapsed, all the doors are closed and the fish is placed in the starting box of the behavioural apparatus. Then, either the left or the right arm is randomly assigned as the location for positive (P) training and a randomly selected full coloured card (green or red) is added to this arm. Pseudo-random selection was used to effectively counterbalance colour allocations across fish. The guillotine door located at the entrance of the P arm is then opened and the fish is allowed to enter the P arm to receive the positive outcome (i.e. social reward; exposure to a conspecific). Conspecifics used as social rewards were juveniles of the same age (sexually undifferentiated) that were housed in a demonstrator tank (5 cm × 5 cm with 8 cm-high side walls) during the judgement bias procedure (approximately 10 min per session). Conspecific rewards were rotated between individuals and did not exhibit abnormal behaviors (such as stress-related or anxiety-like behaviors). The social reward is located at the lateral end of the arm, requiring juveniles to swim to access it, which provide evidence of the rewarding value of the social stimulus. After an additional minute, the social reward is removed, and the fish are allowed to return to the start box on their own. This process is performed 4 consecutive times with 1 min of inter-trial interval (ITI). After this, the session is ended and the juvenile is returned to its isolation tank. The water of the experimental setup is changed between individuals.

The first session of the training phase is performed on day 1 after the habituation phase (inter-phase interval of 4–6 h). The second session of training phase is performed on day 2. During training sessions (day 1 and day 2), juveniles are trained in a discrimination task in which the negative (N) arm (including the second colour cue; green or red) is incorporated to the procedure and fish receive a negative outcome (i.e. punishment) when they approach the end of the N arm. The punishment consists in chasing the fish with a net (different in size and colour than the one used for transferring them between apparatus) for 5 s inside the N arm. Once juveniles enter the N arm, the guillotine door is closed allowing 5 s of chasing without fish leaving the arm. In the training phase, only one door is opened in each trial, either the one of the P (rewarded) or the N (aversive) outcome. Each training session consists of eight entries in total (four negative (N) and four positive (P)) in a pseudo-random sequence (i.e. P P N N P N P N). Before the start of each training session, the fish is placed into the starting box and 1 min of ITI is then applied. Once the ITI has elapsed, the target door is opened and the juvenile has access to the arm. If juveniles do not enter the N arm during a training trial, the guillotine door is left open for 1 min until the negative trial ends. The door is then closed, and the corresponding 1-min ITI is applied. The location (i.e. right or left) and the associated colour cue (i.e. red or green) of the training arms (P and N) are counterbalanced between individuals. Once the sequence of 8 trials is completed the juvenile is returned to its isolation tank and the water of the experimental setup is changed.

The test session is performed on day 3 and consists of 6 pre-training trials (i.e. P P N N P N) followed by the presentation of P and N cue tests. Only one cue option is presented at a time (i.e. no choice), while reinforcements are absent. After P and N testing, the ambiguous cue test (A) is displayed. Each cue test is interspersed with one P and one N outcome presentation (i.e. training trials) since our previous pilot experiments showed that interspersing training trials with cue tests enhance short-term memory performance and shorten the time taken for the fish to learn the task. The full test sequence consists therefore of 13 trials in total, with five P trials, five N trials, and three cue tests (e.g. P P N N P N **P** P N **N** P N **A**; cue testing in bold). The time between the doors being opened and the fish leaving the arm (once the specific outcome was displayed) is recorded for each cue test. Based on previous studies in zebrafish^12,34,60^, we have established an individual criteria for selecting fish that successfully discriminate between P and N stimuli. Juveniles exhibiting < 40 s of difference between P and N latencies are therefore retrospectively excluded from posterior analyses. After the test session, the juvenile is returned to its isolation tank and the water of the experimental setup is changed. After the behavioural test, juveniles that learned to discriminate between N and P testing are scored using a judgement bias score (JBS), which is computed from the latencies (L) to enter the P, N, and A arms for each fish [JBS = (L_A_–L_P_)*100/(L_N_–L_P_)], and used to order individuals in an optimistic/pessimistic axis. The JBS is considered as an index that describes the extent to which each individual responds to the ambiguous cue in a manner similar to the positive or negative cues, controlling therefore for individual motivation. Therefore, a JBS of 50 corresponds to a response latency to the ambiguous cue that lies exactly halfway between the individual’s response latencies to the positive and negative cues. JBSs < 50 suggest a response to the ambiguous cue more similar to the positive cue, while JBSs > 50 suggest a response more similar to the negative cue.

#### Open field test with shelter (OFT)

The OFT, which is based on measures of activity/locomotion and exploration of individuals that are exposed to a novel environment^61–63^, was performed following the procedure as described previously for European sea bass^64^. In this case, a sheltered area was added to the OFT arena. Notably, open field tests with shelter are also used to assess risk-taking behaviour (boldness)^65,66^. In such behavioral tests, fish are presented with a choice between a sheltered area (safe zone) and an open, exposed area (risky zone). Bold fish are more inclined to venture into the risky zone and explore it, even in the presence of potential threats or uncertainties. The behavioural apparatus consists of a square glass tank covered with external white PVC sheets (20 cm × 20 cm with 12-cm-high side walls, Fig. 2A). A closed sheltered area equipped with a transparent guillotine door (10 cm × 10 cm with 7-cm-high side walls) is located in a corner of the arena. The fish is gently placed into the sheltered area for a habituation period of 5 min. Once the 5 min has elapsed, the transparent door is opened and the fish is allowed to explore the arena while is being video-recorded for a period of 20 min. The door remains open during the test. The water is changed between individuals.

#### Novel object test (NOT)

The NOT, which is a standard paradigm used to assess boldness^67,68^ by introducing a novel object into a familiar environment, has been adapted for juvenile European sea bass from the protocol already established for zebrafish^19^. Bold fish are more likely to approach and interact with the novel object, while shy individuals may be more cautious and avoid it. The behavioural apparatus consists of a circular glass tank (21.5 cm of diameter with 9-cm-high side walls) that is surrounded by white wall panels. Each fish is gently placed in centre of the tank for a habituation period of 5 min. The habituation period was selected according to a previous study in zebrafish analysing behavioral responses to novelty in such circular tank^19^. Five minutes after placing the fish in the tank, a novel object (i.e. a blue marble) is placed in the centre of the tank (Fig. 3A). Fish is then video-recorded for 20 min. The water is changed between individuals.

#### Behavioural observations

Video recordings of the behavioural tests (JBT, OFT and NOT) were analysed using multi-event recorder software (Noldus EthoVision XT, Noldus technology, version 12 and The Observer XT, Noldus technology, version 9) and scored blindly to experimental treatment. In the JBT the latency to enter the target arm was scored for each trial test (P, N and A; with a maximum of 120 s). For the OFT, three behavioural outputs were quantified for each fish: (1) latency to leave the shelter for the first time, (2) number of times leaving the shelter, and (3) time spent outside the shelter. For the NOT a region of interest (RoI) near the novel object was defined by a radius of one juvenile body-length from the marble. Six behavioural outputs were quantified for each fish: (1) number of approaches to the novel object, (2) time spent in the novel object zone RoI, (3) distance from the novel object, (4) latency to first approach to the novel object, (5) angular velocity, and (6) time in freezing.

### Experimental design

#### Experiment 1: Behavioural screening and characterization of juvenile sea bass

Sea bass individuals (n = 72) were tested in the judgement bias paradigm, in which individual responses to each treatment (positive, negative, and ambiguous) of the fish that learned the task (n = 43; 59.7% of the tested individuals) were used to categorize them in an optimistic/pessimistic dimension using a judgement bias score (JBS). The day after the JBT was ended, scored individuals were also tested in two additional behavioural assays: first the OFT and then the NOT.

#### Experiment 2: Effect of a pretest unexpected positive event on the response to the ambiguous stimulus

To test whether decision-making under ambiguity is modulated by changes in affective state (Experiment 2) we exposed juveniles to unexpected appetitive experiences before testing them in the evaluation of the ambiguous stimulus in the judgement bias paradigm. Therefore, half of the trained juvenile sea bass received, for the first time, a specimen of *Gammarus* before being exposed to the ambiguous stimulus, while the other half received no pretest positive event. Sea bass juveniles (n = 42) were exposed to the judgement bias paradigm, and those learning the task (n = 30; 71.4% of the tested individuals) were selected for this experiment. The judgement bias test was performed as described above excepting for the presentation of the ambiguous stimulus (A). Before examining individual’s response to A, half of the trained juveniles were randomly selected and received for the first time a pretest appetitive outcome (a specimen of fresh frozen *Gammarus pulex*; Ocean Nutrition; Supplementary Video 3), while the other half of the selected fish received no pretest event. A total of 2 min of ITI was then applied before opening the A arm for both experimental groups. The pretest outcome was dropped after 1 min of ITI when applied. Juveniles that did not consume the pretest outcome were excluded from the experiment. Furthermore, a second set of individuals (n = 36) was trained to receive a positive outcome (i.e. social reward) in the P arm as described in the judgement bias test protocol. Therefore, juveniles learned to perform a Go response (as indicated by a latency < 60 s to enter the arm) when a specific location/colour cue (e.g. left/green; rewarded arm) was presented in order to experience a positive event, while no negative outcomes were displayed. After training, fish were tested with a stimulus that was not associated to the conditioned stimulus (i.e. novel in terms of location and colour; e.g. right/red; unknown arm). As previously described, half of the trained juveniles that learned the task (n = 24; 66.7% of the tested individuals) were randomly selected and received for the first time a pretest positive event, while the other half received no pretest positive event.

### Statistical analyses

The following statistical tests were run in R (v.4.2.2)^69^ and SigmaStat (v.3.5). Effect size estimates (Cohen’s d or partial eta-squared) and 95% confidence intervals (CI) were calculated with the R software package “effectsize”^70^. Outliers were identified for each condition using the generalized extreme studentized deviate procedure with a *p* = 0.05 and a maximum number of outliers of 20% of sample size. Outliers were detected and removed from OFT (a total of 2 outliers) and NOT (a total of 4 outliers) metrics. No outliers were removed from JBT data. The comparisons of the different behavioral metrics derived from OFT and NOT (Experiment 1) between optimistic and pessimistic juveniles were independently performed with Mann–Whitney rank-sum test or unpaired t-test and, consequently, no adjustment for multiple statistical testing was applied. When parametric assumptions were verified, on raw or transformed data, we used the parametric test. When, even after transforming data, parametric assumptions were not met, the non-parametric statistic was used.

The unrotated principal component analysis (PCA; Experiment 1) was performed using the R package “factoextra”^71^ to reduce the number of variables measured in the JBT, OFT and NOT tests to a set of principal components (PCs) that represent linear combinations of the original variables, conserving the maximal data variation.

For the analysis of the effect of unexpected appetitive experiences on the response to the ambiguous stimulus of the judgement bias assay (Experiment 2), we used the R software packages ‘afex’^72^ and ‘lme4’^73^ for the linear mixed-effects models (GLMMs), and the ‘emmeans' package^74^ for planned multiple comparisons (Tukey HSD). The response variables were the latencies to respond to stimuli, that is, the time it took the fish to enter the experimental arms (positive (P), ambiguous (A), negative (N) of the behavioral apparatus). Latencies were restricted to the interval between 0 and 120 s and were log-transformed. The fixed effects were stimuli (with three groups: P, N, A) and treatment (with two groups: control and unexpected positive event). The random effect was the fish identity, since the same individuals were tested in all stimuli for each treatment group (repeated measures). The effect of unexpected positive events on novel stimuli was also evaluated using linear mixed models with fish identity as a random effect. The fixed effects were stimulus (with two groups: training and novel) and treatment (with two groups: control and unexpected positive event). Planned multiple comparisons were then used to evaluate the effect of the interaction of stimulus and treatment. Inspection of model residuals showed satisfactory normal distributions. All *p*-values are two-tailed. The effect of unexpected appetitive events on the JBS was performed with unpaired t-test.

### Supplementary Information

Supplementary Information 1.

Supplementary Information 2.

Supplementary Video 1.

Supplementary Video 2.

Supplementary Video 3.

## Data Availability

Data is made available as supplementary material to the manuscript.
