# Supplementary material for: Unexpected appetitive events promote positive affective state in juvenile European sea bass
Source: Sci Rep. 2023 Dec 12;13:22064. doi: 10.1038/s41598-023-49236-5 (PMC10716175; doi:10.1038/s41598-023-49236-5)
Supplement: Supplementary file 2 — Supplementary Information 2. [file 41598_2023_49236_MOESM2_ESM.docx]

**Supplementary material: Unexpected appetitive events promote positive affective state in juvenile European sea bass**

María V. Alvarado, Alicia Felip, Felipe Espigares, and Rui F. Oliveira

**
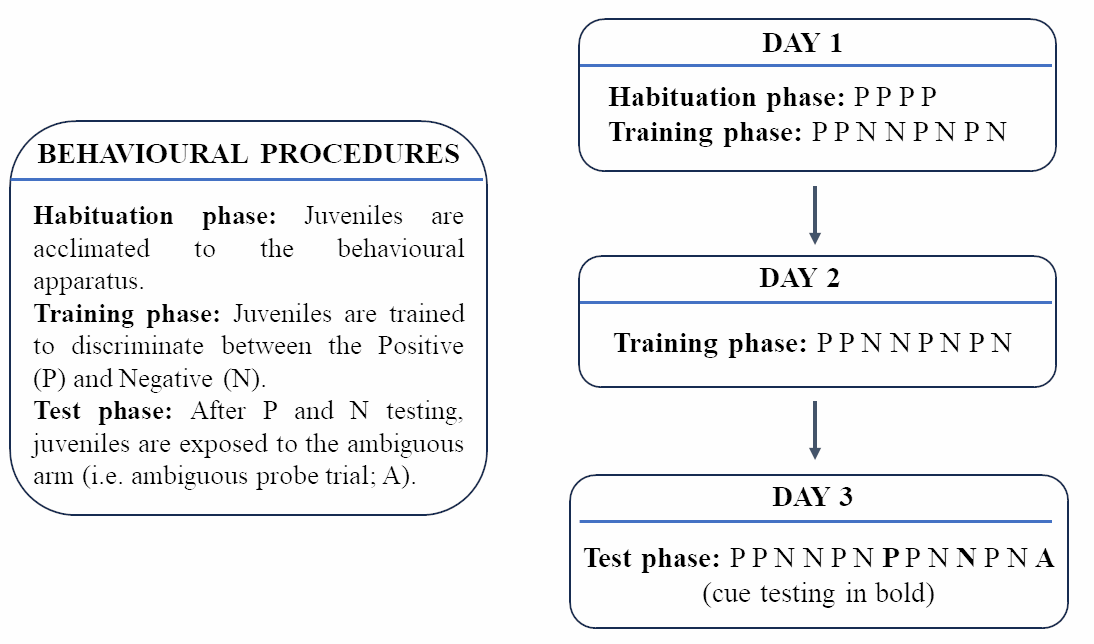
Supplementary figures**

**Figure S1.** Schematic representation of the judgement bias assay in juvenile European sea bass depicting the sequence of the different behavioural procedures.

**
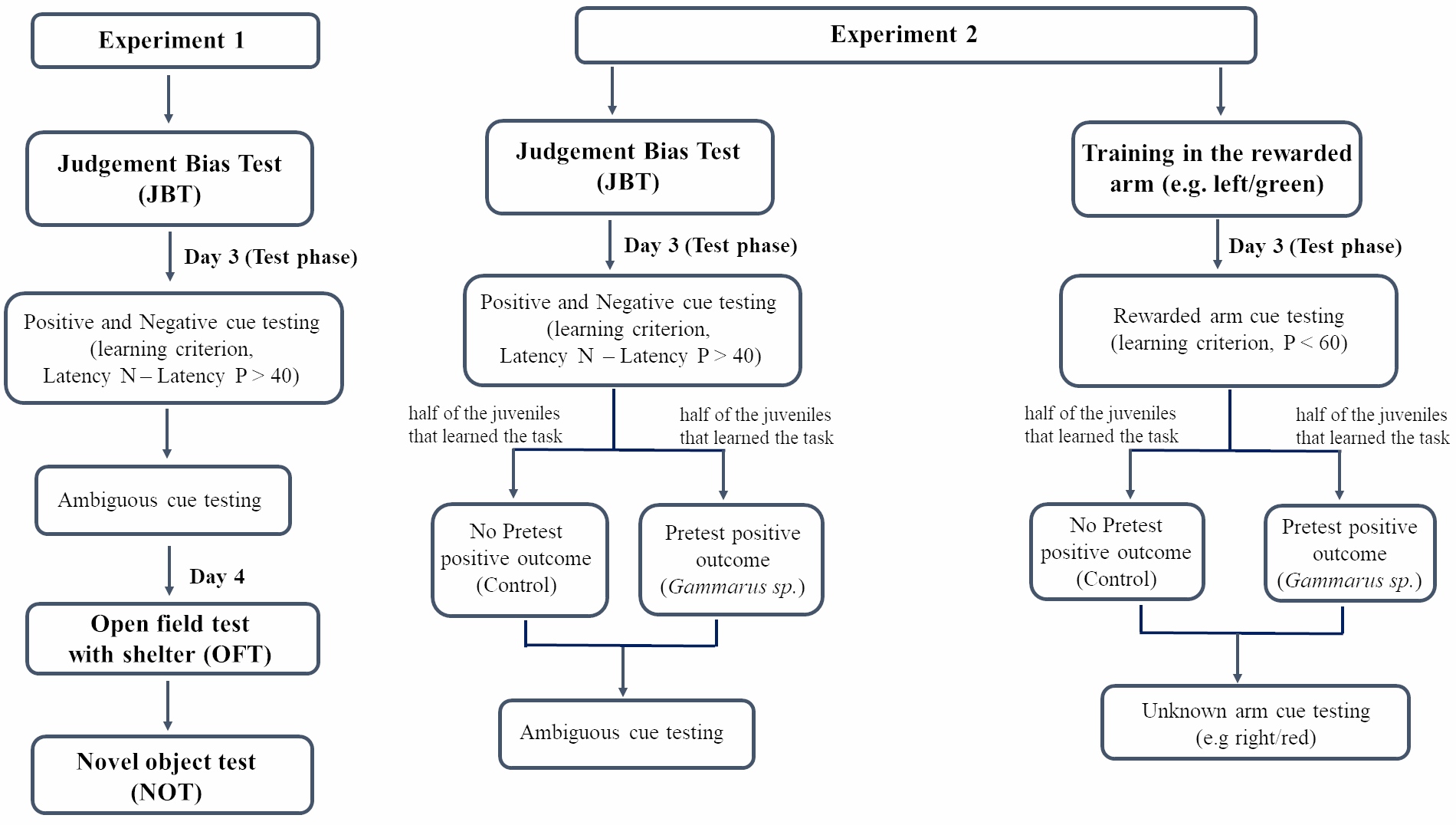
Figure S2.** Schematic representation of the two experiments carried out in this study depicting the sequence of the different behavioural events.
